# Committed to Success: A Structured Mentoring Program for Clinically Oriented Physicians

**DOI:** 10.1016/j.mayocpiqo.2024.05.002

**Published:** 2024-06-14

**Authors:** Nathan Houchens, Latoya Kuhn, David Ratz, Grace L. Su, Sanjay Saint

**Affiliations:** a Medicine Service, Veterans Affairs Ann Arbor Healthcare System, Ann Arbor, MI; b Division of Hospital Medicine, Department of Medicine, University of Michigan, Ann Arbor; c Division of Gastroenterology and Hepatology, Department of Medicine, University of Michigan, Ann Arbor

## Abstract

**Objective:**

To examine impacts of a structured mentorship committee program on academic promotion and participant perceptions because impacts of formal mentorship programs for clinical faculty are unknown.

**Participants and Methods:**

This prospective cohort study at a Midwestern Veterans Affairs tertiary care system from December 17, 2019 to December 31, 2022 included clinical track faculty in the Medicine Service below the rank of Clinical Associate Professor. Mentoring meetings (mentee, committee chair, and mentors) were generally held twice annually. All participants were surveyed after each meeting (response rate: 100%).

**Results:**

All 23 of 23 (100%) eligible faculty were enrolled as mentees, and 49 distinct meetings occurred. Three (13%) mentees were promoted, and the remaining 20 (87%) continued in the program. Mean scores (SD), scaled 1 (strongly disagree) to 5 (strongly agree), for mentors and mentees were 4.71 (0.51) and 4.80 (0.54) for “effective use of my time”; 4.58 (0.64) and 4.37 (0.49) for “appropriate progress since last meeting”; 4.52 (0.66) and 4.31 (0.64) for “program increased my work satisfaction”; and 4.07 (0.96) and 3.75 (0.92) for “program reduced my work burnout,” respectively.

**Conclusion:**

Clinically oriented physicians viewed the program positively. It appeared to help junior faculty get promoted and led to improved work satisfaction and reduced burnout.

Mentorship is an integral aspect in the successful careers, professional development, and growth for many physicians.^1^ Defined as “a dynamic, reciprocal relationship in a work environment between an advanced career incumbent (mentor) and a beginner (mentee), aimed at promoting the development of both,”^2^ mentorship has long existed as a core component for faculty in academic medicine, but its importance has only more recently been described. Mentoring relationships may provide several well-defined benefits to the mentor, mentee, and institution including effective selection of future career, faster and greater likelihood of academic promotion, increased scholarly productivity and funding, improved faculty retention, and enhanced work satisfaction.^3^, ^4^, ^5^, ^6^, ^7^, ^8^, ^9^, ^10^, ^11^, ^12^, ^13^, ^14^ Conversely, lack of or inadequate mentorship is noted to be one of the most negative influences on career progression in medicine.^15^^,^^16^ Professional satisfaction^17^ is even more relevant recently because rates of burnout have been shown to be increasing.^18^

Historical styles and models of mentorship have resulted in several challenges. The traditional mentoring relationship is dyadic between a mentor and mentee and is, by definition, hierarchical.^12^ Several potential pitfalls exist within this type of relationship including disparate goals and levels of commitment, conflicts of interest, and generational tensions.^15^^,^^19^, ^20^, ^21^, ^22^ As a result, other models of mentorship have been suggested such as groups of peers or series of mentors from different career stages or domains.^23^ Some have suggested these newer models might overcome the drawbacks of dyadic mentoring relationships and allow for a diverse network of mentors possessing differing competencies the mentee wishes to attain.^24^, ^25^, ^26^ In addition, formal mentorship has mostly focused on academic medical centers and been associated with junior physician-scientist faculty whose careers focus primarily on garnering extramural research support and generating scholarly output in the form of peer-reviewed articles and whose markers of success are objective and relatively easily measured.^27^ In a previous prevalence study, physician-scientists were more likely to self-report receipt of mentorship than clinician-educators.^28^ Yet, the benefits of mentorship are broadly applicable, including to those junior faculty who primarily engage in direct patient care and clinical teaching. Little scholarship has examined direct impacts of formalized mentorship programs for clinically oriented faculty on important outcomes like burnout and professional satisfaction.

We thus established a structured clinician mentoring program for faculty members with a clinical focus. Given the importance of mentorship for career success and satisfaction, our objective was to describe the development of a formalized mentorship committee program and explore its impacts on academic advancement and a myriad of participant perceptions including value assessment of mentoring meetings, work satisfaction, and burnout.

## Participants and Methods

### Study Design and Setting

This prospective cohort study examined the impacts of the clinician mentoring program on academic promotion and participant perceptions in a variety of domains. The program was established in 2019 in the medicine service at a Midwestern Veterans Affairs (VA) health care system, operating as a tertiary referral medical center for Veterans. This VA is closely affiliated with a quaternary research-intensive academic medical center. All medicine service physicians at this VA have faculty appointments at the affiliated academic medical center. Data collection took place for 3 years (December 17, 2019 to December 31, 2022) after the inception of the program.

### Participants

All clinical track faculty members in the medicine service who had at least 62.5% VA effort and who were below the academic rank of Clinical Associate Professor were invited to participate through email invitations sent by an author (N.W.H.). This email included a program overview document containing a description, goals, and guiding principles of the program (Supplemental Appendix A, available online at http://www.mcpiqojournal.org), eligibility criteria, proposed time commitment, elicitation of interest, and initial questions to encourage the faculty member to consider potential mentors. Faculty meeting these criteria who had existing mentoring committees focused only on research were also included. Junior faculty on the tenure track were excluded. The identified project manager (L.E.K.) sent at least 2 follow-up emails to faculty if they did not respond to the initial inquiry. Recruitment for the program occurred on a rolling basis such that new faculty meeting eligibility criteria and existing faculty who become eligible are invited.

For each mentee, a mentoring committee was established. These committees comprised between 3 and 6 committee members and 1 committee chair, chosen by the mentee and their advisors. Mentees often requested mentor involvement through email communications that included a copy of the program overview, the request, and proposed time commitment. The committee chair was often a section or division chief. Other mentors who served on committees included leaders in the health care system, research, quality improvement, education, policy, or any other domain in which the mentee was interested. Committees disbanded once the mentee reached the level of Clinical Associate Professor, reduced their VA appointment to less than 62.5%, or left the academic affiliate.

### Procedures

Program support was provided by an experienced project manager (L.E.K.). This individual served as the program coordinator and was instrumental in its success. She contributed approximately 15 hours monthly to complete a broad array of tasks (eg, scheduling and sending reminders for each meeting, distributing handouts, and answering mentees’ questions). The project manager’s effort was primarily provided by the VA’s Chief of Medicine and through the local VA Health Systems Research organization. Support for and commitment to the program was also widespread among institutional leadership at multiple levels, including the VA Chief of Staff who enrolled as a mentee.

Mentoring committee meetings generally occurred twice per year for 1 hour at a time held in person or via a virtual meeting platform. Preparation ensured that the meetings were as successful and useful as possible. The mentee was primarily responsible for arranging and running the meetings. However, the project manager tracked participation and engagement, coordinated communications, helped to schedule meetings, and assisted with all meeting materials, including distribution of program information and an updated copy of the mentee’s curriculum vitae to all committee members for review before the meeting. The mentee was expected to be familiar with key resources, including general guidance for effective menteeship^29^ as well as clinical track promotional information and criteria for the academic affiliate. During committee meetings, the mentee and chair guided conversation that spanned the mentee’s short-term, mid-term, and long-term goals, assessment of personal and professional satisfaction with the mentee’s current role, updates in the key areas in which the mentee spent time, suggestions on career advancement strategies, and feedback on how the committee could most effectively mentor and sponsor the mentee.

### Measurements

At the conclusion of each mentoring committee meeting, we distributed a standardized survey instrument called the Mentoring Meeting Assessment Tool (MMAT) (Supplemental Appendix B, available online at http://www.mcpiqojournal.org). This electronic, Likert scale–based survey had been developed by program leadership through creation and revision of questions after receiving feedback from team members and departmental leadership. It was finalized approximately 9 months after the first mentoring committee meeting and was subsequently sent via email to all participants including mentee, chair, and committee members after each meeting. The MMAT elicited information on logistics of the meeting (eg, the meeting started on time, the mentee’s curriculum vitae was distributed before the meeting, and the meeting was a good use of time); perceptions of effectiveness (eg, how feedback was given and received); clarity of next steps; assessment of mentee progress since the last meeting; and impacts of the mentoring program on satisfaction and burnout. Participants completed only questions that were appropriate to their respective roles (ie, mentor/chair or mentee). The project manager followed-up with all mentors and mentees to ensure responses were collected.

The primary outcome of participant perceptions was the calculated mean Likert score of the individual questions, with higher scores representing more agreement with the statement. Participant reflections were also collected through free-text response on the survey instrument. Promotion to the level of Clinical Associate Professor was also tracked.

### Statistical/Data Analyses

Descriptive statistics were used to characterize the population and assessment tool. Meeting assessment response data were collected directly from meeting attendees via Qualtrics survey software. For comparisons made between mentees and mentors (where the same question was asked to both), we used a mixed-effects linear regression model with random intercepts for both the committee and the individual to account for correlation both within the committee and within repeated measures on the same individual. The 2-tailed α was set at .05. Analyses were conducted using Stata/MP 17.0.

## Results

The clinician mentoring program enrolled 23 mentees of 23 total eligible faculty members (100%) from 10 unique clinical sections in which 49 distinct 1-hour meetings occurred. Number of meetings per committee ranged from 1 to 4. Overall response rate to the MMAT from all participants was 100%. During the study period, 3 of the 23 (13%) mentees were promoted to the rank of Clinical Associate Professor, and 20 of 23 (87%) continued in the program.

Total numbers of MMAT responses differed because some questions were added to the assessment tool after initiation of data collection, and some (eg, the mentee has made appropriate progress since the last meeting) were only applicable to those meetings after the first one. With respect to mentors, 98% of responses agreed (agree or strongly agree) that committee meetings were an effective use of their time, 99% agreed that feedback provided was received by the mentee in a positive manner, 94% agreed that next steps were clear, 94% agreed that the mentee had made appropriate progress since the prior meeting, and 91% and 63% agreed that the mentoring program increased their work satisfaction and reduced their level of burnout, respectively. With respect to mentees, 98% of responses agreed that committee meetings were an effective use of their time and that they were comfortable raising issues with their mentors, 90% agreed that next steps were clear, and 91% and 56% agreed that the mentoring program increased their work satisfaction and reduced their level of burnout, respectively. All mentee responses (100%) agreed that feedback provided was specific, actionable, and focused on how to improve, that they trust that mentors are committed to their professional success, that mentors are helping them set and achieve career goals, and that they had made appropriate progress since the previous meeting.

Mean scores from 1 (strongly disagree) to 5 (strongly agree) with SDs are reported. Among mentors, the domain with the highest score was “feedback was received in a positive manner by the mentee,” with a mean score of 4.85 (SD=0.40). Among mentees, the domain with the highest score was “I trust that mentors are committed to my success,” with a mean score of 4.94 (SD=0.24). The lowest mean scores among mentors and mentees were in the domain of “the program reduced my work burnout” at 4.07 (SD=0.96) and 3.75 (SD=0.92), respectively. For identical questions posed to both parties, comparisons of responses between mentors and mentees yielded no significant differences (“effective use of my time,” *P*=.39; “next steps were clear,” *P*=.11; “program increased my work satisfaction,” *P=*.15; “program reduced my work burnout,” *P=*.09). Perceptions of the program and its impacts on participants collected from both mentors and mentees are found in Table 1, and selected participant comments are found in Table 2.

**Table 1:** Mentor and Mentee Perceptions of the Clinician Mentoring Program^a^

	Strongly disagree, n (%)	Disagree, n (%)	Neither agree nor disagree, n (%)	Agree, n (%)	Strongly agree, n (%)	Mean (SD)
Mentors						
Effective use of my time (n=188)	0 (0)	1 (<1)	2 (1)	48 (26)	137 (73)	4.71 (0.51)
Feedback was received in a positive manner by mentee (n=188)	0 (0)	1 (<1)	0 (0)	25 (13)	162 (86)	4.85 (0.40)
Next steps were clear (n=188)	0 (0)	0 (0)	11 (6)	57 (30)	120 (64)	4.58 (0.60)
Mentee has made appropriate progress since the last meeting (n=99)^b^^,^^c^	0 (0)	1 (1)	5 (5)	29 (29)	64 (65)	4.58 (0.64)
Program increased my work satisfaction (n=113)^b^	0 (0)	0 (0)	10 (9)	34 (30)	69 (61)	4.52 (0.66)
Program reduced my work burnout (n=113)^b^	0 (0)	3 (3)	39 (35)	18 (16)	53 (47)	4.07 (0.96)
Mentees						
Effective use of my time (n=49)	0 (0)	1 (2)	0 (0)	7 (14)	41 (84)	4.80 (0.54)
Comfortable raising issues with mentors (n=49)	0 (0)	0 (0)	1 (2)	12 (25)	36 (73)	4.71 (0.50)
Feedback was specific, actionable, and focused on how to improve (n=49)	0 (0)	0 (0)	0 (0)	14 (29)	35 (71)	4.71 (0.46)
Trust that mentors are committed to my success (n=49)	0 (0)	0 (0)	0 (0)	3 (6)	46 (94)	4.94 (0.24)
Mentors are helping me set and achieve career goals (n=49)	0 (0)	0 (0)	0 (0)	8 (16)	41 (84)	4.84 (0.37)
Next steps were clear (n=49)	0 (0)	2 (4)	3 (6)	15 (31)	29 (59)	4.45 (0.79)
I have made appropriate progress since the last meeting (n=27)^b^^,^^c^	0 (0)	0 (0)	0 (0)	17 (63)	10 (37)	4.37 (0.49)
Program increased my work satisfaction (n=32)^b^	0 (0)	0 (0)	3 (9)	16 (50)	13 (41)	4.31 (0.64)
Program reduced my work burnout (n=32)^b^	0 (0)	2 (6)	12 (38)	10 (31)	8 (25)	3.75 (0.92)

a Abbreviation: SD, standard deviation.

b Question added to the assessment tool after initiation of the program.

c Question asked only after the initial meeting.

**Table 2:** Selected Comments From Program Participants

From mentors
“This is a wonderful program—should continue and hopefully is being emulated in other departments.”
“Dr. [redacted] continues to make terrific strides in quality improvement, medical education, research, and clinical care. She rightly brought up issues of inadequate employees which limits our ability to care for Veterans needing renal transplantation. She sought advice on how to deliver feedback effectively. We discussed opportunities for her to get national meeting exposure which will bolster her promotion packet when the time comes.”
“Valuable experience for mentee to gain variety of perspectives on career development and mentors to learn from each other regarding mentoring styles and advice.”
“It’s wonderful hearing the candid advice as well as obvious caring support of [redacted] from the members of this meeting.”
From mentees
“Excellent feedback and forum for discussion. I do feel a strong commitment by committee to my success. I feel comfortable disclosing difficult issues and asking for help with difficult questions.”
“It was so encouraging to have the opportunity to hear input from so many phenomenal clinicians and researchers and to have them all in the room at the same time. Great resource of information and very uplifting they all gave their time.”
“The meeting was very helpful in channeling and focusing my efforts to achieve my career goals.”
“I have a diverse mentorship committee in terms of their background of expertise, and it is has been very helpful to hear their feedback and perspectives. I think all of them are great advocates on my behalf and as I continue in my career, I think they will be great mentors, sponsors and coaches.”
“Really useful to have the carved out space to talk about bigger picture career directions rather than just the nuts and bolts of research. I appreciate the more strategic discussions we have in these meetings.”
“Working through the agenda was a valuable use of time and important self-reflection exercise. The meeting was valuable with suggestions regarding how to address constructive learner feedback, increase teaching effectiveness, improve scholarly activity, and general career advice.”

## Discussion

In this prospective cohort study spanning 3 years at a large Midwestern academic medical center, we report the impacts on, perceptions of, and academic advancement rates for 23 clinical track faculty participants in a clinician mentoring program. We found that mentees, committee chairs, and mentors were highly satisfied with the process of mentorship in the committee structure, that the majority felt the program enhanced their work satisfaction and reduced their levels of burnout, that all eligible enrolled mentees continued in the program, and that approximately 1 in 8 mentees were promoted to the academic rank of Clinical Associate Professor. Taken together, these findings suggest that a structured mentoring program is well received and successful at achieving the goal of elevating its members’ personal and professional satisfaction.

Our study adds to the growing literature surrounding mentorship in academic medicine. Many previous studies and systematic reviews have examined the prevalence, models, benefits, and potential pitfalls of mentoring. In one study, fewer than half of academic physicians reported receipt of mentorship during their careers.^8^^,^^25^ Our clinician mentoring program was designed to ensure that all clinicians, including those clinical track faculty who have historically not been included, could receive mentorship. A variety of mentoring models exist including dyadic (both single and multiple), team or network, peer, facilitated peer, distance, functional, and speed styles of mentoring.^5^^,^^8^ Recognizing that a single mentor is unlikely to meet the myriad needs and wants of the mentee,^22^^,^^30^ our program used a committee or team-based structure to create a hybrid model in which the mentee, with some guidance if desired, chose mentors on the basis of their individual interests and career goals. In many committees, these mentors included peers. This approach, similar to the mosaic model by Welch et al,^31^ which combined vertical and peer mentoring,^31^ allowed a diversity of perspectives with a common goal of supporting and sponsoring the mentee. Our program did not provide incentives to mentors to participate^32^ nor was there specific training required for mentors.^33^^,^^34^ Despite this, mentors reported that mentoring committee meetings were an effective use of their time, increased their work satisfaction, and reduced their burnout.

Relationships between mentors and mentees appear to play an integral role in the potential for burnout mitigation. This is believed to be due to a stronger personal connection, sharing of psychosocial support, and a better ability to align professional activities with those that are fulfilling and meaningful to the mentee.^35^ When examining the impacts mentorship and coaching have on burnout, however, previous studies at various levels of health care trainee or clinician demonstrate mixed results, with some showing improved burnout for the mentee^36^, ^37^, ^38^, ^39^ or both mentee and mentor^40^ and others showing no effect^41^^,^^42^ or disparate changes in burnout for different groups.^43^ Our study reveals an important positive effect on wellness and burnout reported by both mentors and mentees. It is unknown what aspect or aspects of our program led to this improvement, but perhaps it is the mentee’s agency in choosing their respective committee members that fosters high-quality mentoring relationships and leads to a greater likelihood of enhanced work satisfaction and reduced burnout.

Several strengths distinguish our study from other literature related to mentorship. We used a standardized survey instrument that allowed direct comparisons between mentors and mentees in a variety of domains. This technique allowed real-time assessments from all participants of the effectiveness of mentoring relationships within the program. In addition, of all eligible faculty, 100% were enrolled into the program and continued to participate, and the response rate to the MMAT was 100%. This universal adoption and engagement ensured an accurate and representative data set for our faculty. There was specialty diversity in faculty enrolled, with 10 distinct clinical sections represented. Finally, we used a single “centralized” project manager who helped to coordinate meetings, distribute important information before meetings, and facilitate gathering of programmatic feedback. This allowed the mentee and committee members to focus on core elements of giving and receiving mentorship rather than meeting logistics. Given the program’s success, it has more recently been offered to those faculty who have 50% VA effort or more as well as to faculty in services outside of medicine (eg, ambulatory care and anesthesiology).

Our study should also be interpreted in the context of several limitations. First, 3 years of follow-up were inadequate to completely characterize long-term effects of mentorship including academic advancement and other professional opportunities. For instance, faculty in our institution spend an average of approximately 5 years in the rank of Clinical Assistant Professor before consideration for promotion, and as such, our rates of academic promotion may be underestimated. Second, although all eligible faculty were enrolled, mentee engagement with the program was variable. For instance, one mentee may have held regular meetings twice a year or more frequently, whereas another may have only participated once annually. As such, there was likely selection bias introduced if, for example, more enthusiastic mentees were more often represented in the data. In addition, rolling recruitment methodology and varying time frames of individuals’ enrollment in the program contribute to challenge interpreting discrete numbers of meetings per committee. Third, the MMAT instrument was finalized and implemented 9 months after the mentoring program was established. Moreover, some questions were added only after data collection began. Thus, our MMAT data are incomplete, particularly for responses obtained in the program’s infancy. Finally, our study focused exclusively on academic advancement and subjective participant perceptions, which are only a subset of possible outcomes from mentoring relationships. We did not capture other specific, objective outcomes relevant to professional growth and development such as speaking opportunities, teaching awards, and leadership roles. Metrics such as tenure, scholarly publications, and grant submissions are more relevant for research-oriented clinicians (ie, tenure track) and fall outside the scope of this mentorship program’s outcomes. However, there is another formal mentoring program, established at our institution and focused on research, which tracks these outcomes.

## Conclusion

A formalized mentoring program harnessing committee structures and targeted to physicians on the clinical track at a research-intensive medical school appeared to be successful in helping junior faculty get promoted. The program was viewed positively by both mentees and their mentorship committee members. In fact, for many participants, the program led to enhanced work satisfaction and reduced burnout. Future research should focus on examination of additional mentee achievements (eg, teaching awards and leadership positions), which are likely to be as varied as the mentees and their interests. Our findings carry important implications for how hospitals and academic health care systems might consider establishing formal mentoring programs for clinically oriented faculty to foster their professional development.

## Potential Competing Interests

The authors report no competing interests.
