# Supplementary material for: Committed to Success: A Structured Mentoring Program for Clinically Oriented Physicians
Source: Mayo Clin Proc Innov Qual Outcomes. 2024 Jun 14;8(4):356–63. doi: 10.1016/j.mayocpiqo.2024.05.002 (PMC11225678; doi:10.1016/j.mayocpiqo.2024.05.002)
Supplement: Supplemental Appendix A [file mmc1.pdf]

Appendix A. Clinician Mentoring Program description

VA CLINICIAN MENTORING PROGRAM

[AUTHOR]
[AUTHOR]
[AUTHOR]

Project Manager: [AUTHOR]

Background: [VA NAME] is committed to mentoring junior faculty. To this end, the [VA NAME] Mentoring Program was developed. The program's goal is to have every clinical track faculty member in the Medicine Service at [VA NAME] (who is 5/8ths VA or more and below the rank of Clinical Associate Professor) establish and maintain a mentoring committee. Junior faculty on the instructional track will continue to have mentoring committees coordinated by their respective [UNIVERSITY AFFILIATE] Division Chiefs.

Committee Composition: The total number of committee members will usually be between 3 and 6. The chair of the mentoring committee will usually be the VA Section Chief or another faculty member selected by the mentee. Other members of the committee will consist of [UNIVERSITY AFFILIATE] Division/Department leaders (e.g., Division Chief, Vice Chair), [VA NAME] Medicine Service leadership (e.g., Chief of Medicine, Associate Chief of Medicine), [VA NAME] leadership (e.g., Chief of Staff, Associate Chief of Staff, Chief of another service such as Surgery, Ambulatory Care, or Mental Health), and other appropriate individuals identified by the mentee and chair of the mentoring committee.

Overall Logistics: Meetings will occur at least once or twice per year, for 1-hour at a time. The meetings will be held in-person or via Zoom. Arranging, coordinating, and running the meetings will be the responsibility primarily of the mentee. [AUTHOR], project manager, is available to provide scheduling assistance and can be reached at [EMAIL].

Mentee Resources: Once enrolled in the program, the mentee should become familiar with the following key resources.

- BMJ article (the four golden rules of effective menteeship): https://www.bmj.com/content/354/bmj.i4147.full
- [UNIVERSITY AFFILIATE CLINICAL TRACK PROMOTION REQUIREMENTS]

Committee Meeting Logistics:
At each mentorship meeting, the mentee will be asked to:

1) Send a copy of their updated CV to all committee members at least 1 week before the meeting date.
2) Be prepared to discuss their short-term goals (within 1 year), mid-term goals (2-3 years), and long-term goals (> 3-5 years) with 1-3 specific goals they hope to accomplish for each time period.
3) Provide the committee with an overall assessment of how satisfied they are

with their current role and responsibilities.

4) Provide the committee with an update in each of the key domains in which they are spending time: education, clinical work, scholarly activity, and leadership roles (for education, the mentee will be expected to review their trainees' evaluations beforehand and summarize for the committee). Ideally, the mentee would provide a self-appraisal of how things are going in each domain; the committee can then provide comments and guidance to help them reach their fullest potential.
5) Provide the committee with feedback on how they can help the mentee become as effective a faculty member as possible. The mentee is encouraged to bring up questions or thorny issues that they are grappling during committee meetings.

Tracking and Completion: Medicine Service will track mentoring committee meeting dates through the VA Section Chiefs, committee chairs, and/or mentees; [AUTHOR] will be the Medicine Service representative who will help keep track of this program. The committee will stop meeting and disband once the mentee reaches the level of Associate Professor (or above), reduces their VA appointment to less than 5/8ths, or leaves [UNIVERSITY AFFILIATE].
