# Supplementary material for: Committed to Success: A Structured Mentoring Program for Clinically Oriented Physicians
Source: Mayo Clin Proc Innov Qual Outcomes. 2024 Jun 14;8(4):356–63. doi: 10.1016/j.mayocpiqo.2024.05.002 (PMC11225678; doi:10.1016/j.mayocpiqo.2024.05.002)
Supplement: Supplemental Appendix B [file mmc2.pdf]

Appendix B. Mentoring Meeting Assessment Tool

Mentoring Meeting Assessment Tool (MMAT)

1. Please enter your name ___________________________

2. Please enter your role (select only one option)
    - ☐ Mentee
    - ☐ Mentoring committee chair
    - ☐ Mentoring committee member

3. Please enter the mentee's name (MENTORS ONLY) ____________________

4. Please enter the date of the meeting (MENTEES ONLY) ________________

5. Mentee's CV was sent to all mentors before the meeting (MENTORS ONLY)
    - ☐ Yes
    - ☐ No

6. The meeting began on time
    - ☐ Yes
    - ☐ No

7. Sufficient time was allotted for the meeting
    - ☐ Strongly agree
    - ☐ Agree
    - ☐ Neither agree nor disagree
    - ☐ Disagree
    - ☐ Strongly disagree

8. Overall, the meeting was an effective use of my time
    - ☐ Strongly agree
    - ☐ Agree
    - ☐ Neither agree nor disagree
    - ☐ Disagree
    - ☐ Strongly disagree

9. I discussed _____ action items I wanted to during the meeting (MENTEE ONLY)
    - ☐ All
    - ☐ Most
    - ☐ Some
    - ☐ Few
    - ☐ No

10. I felt comfortable raising issues with mentors (MENTEE ONLY)
    - ☐ Strongly agree

- ☐ Agree
- ☐ Neither agree nor disagree
- ☐ Disagree
- ☐ Strongly disagree

11. Feedback given by mentors was specific, actionable, and focused on how to improve (MENTEE ONLY)
    - ☐ Strongly agree
    - ☐ Agree
    - ☐ Neither agree nor disagree
    - ☐ Disagree
    - ☐ Strongly disagree

12. Feedback given by mentors was received in a positive manner by mentee (MENTORS ONLY)
    - ☐ Strongly agree
    - ☐ Agree
    - ☐ Neither agree nor disagree
    - ☐ Disagree
    - ☐ Strongly disagree

13. I trust that my mentors are committed to my professional success (MENTEE ONLY)
    - ☐ Strongly agree
    - ☐ Agree
    - ☐ Neither agree nor disagree
    - ☐ Disagree
    - ☐ Strongly disagree

14. My mentors are helping me set and achieve career goals (MENTEE ONLY)
    - ☐ Strongly agree
    - ☐ Agree
    - ☐ Neither agree nor disagree
    - ☐ Disagree
    - ☐ Strongly disagree

15. At the end of the meeting, next steps were clear (i.e., who is doing what by when)
    - ☐ Strongly agree
    - ☐ Agree
    - ☐ Neither agree nor disagree
    - ☐ Disagree
    - ☐ Strongly disagree

16. I have made appropriate progress since the last mentoring committee meeting (MENTEE ONLY)
    - ☐ Strongly agree
    - ☐ Agree

- ☐ Neither agree nor disagree
- ☐ Disagree
- ☐ Strongly disagree

17. The mentee has made appropriate progress since the last mentoring committee meeting (MENTORS ONLY)
    - ☐ Strongly agree
    - ☐ Agree
    - ☐ Neither agree nor disagree
    - ☐ Disagree
    - ☐ Strongly disagree

18. Overall, this mentoring program has increased my level of satisfaction with my work
    - ☐ Strongly agree
    - ☐ Agree
    - ☐ Neither agree nor disagree
    - ☐ Disagree
    - ☐ Strongly disagree

19. Overall, this mentoring program has reduced my level of burnout (e.g., exhaustion, depersonalization, and/or reduced achievement) from my work
    - ☐ Strongly agree
    - ☐ Agree
    - ☐ Neither agree nor disagree
    - ☐ Disagree
    - ☐ Strongly disagree

Please provide additional comments:

